# Supplementary figures and images for: RAS–p110α signalling in macrophages is required for effective inflammatory response and resolution of inflammation
Source: eLife. 2025 Apr 24;13:RP94590. doi: 10.7554/eLife.94590 (PMC12021417; doi:10.7554/eLife.94590)

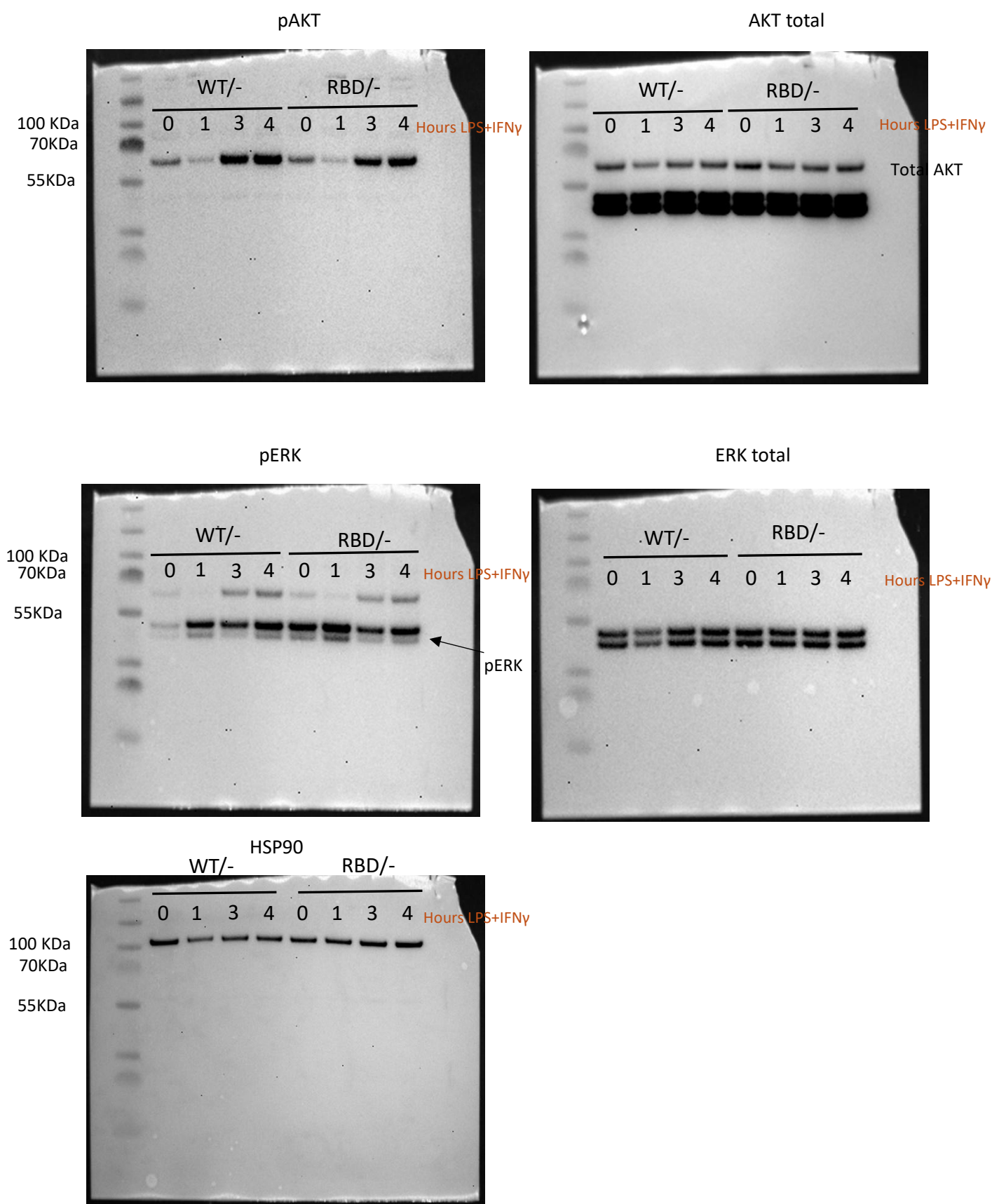

Figure 1A, source data 1: Original membranes corresponding to Figure 1, panel A.

Supplement: Figure 1—source data 1. [file elife-94590-fig1-data1.zip › Figure 1A-source data 1.pdf]

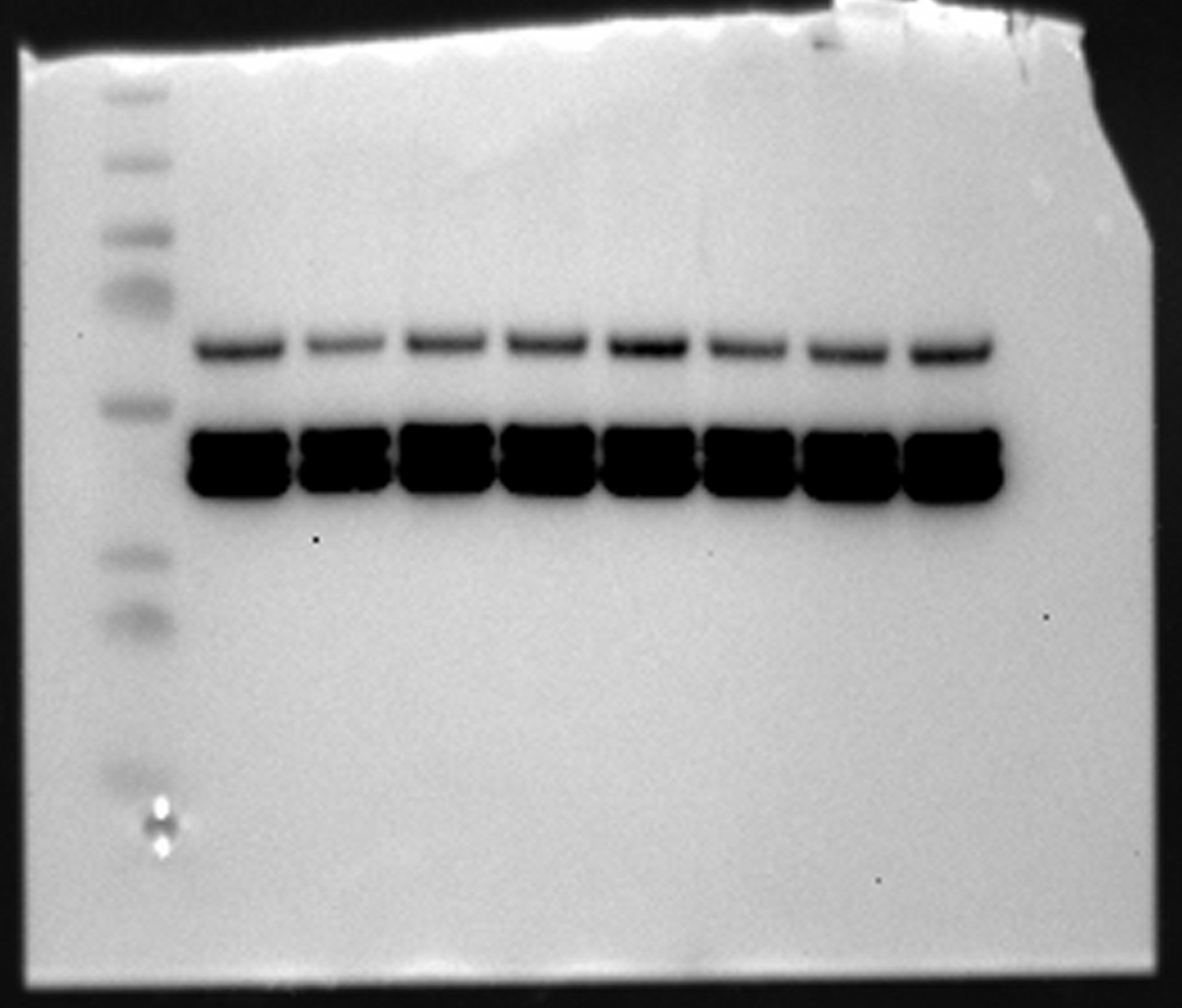

Supplement: Figure 1—source data 2. [file elife-94590-fig1-data2.zip › Figure 1A/akt total mb.jpg]

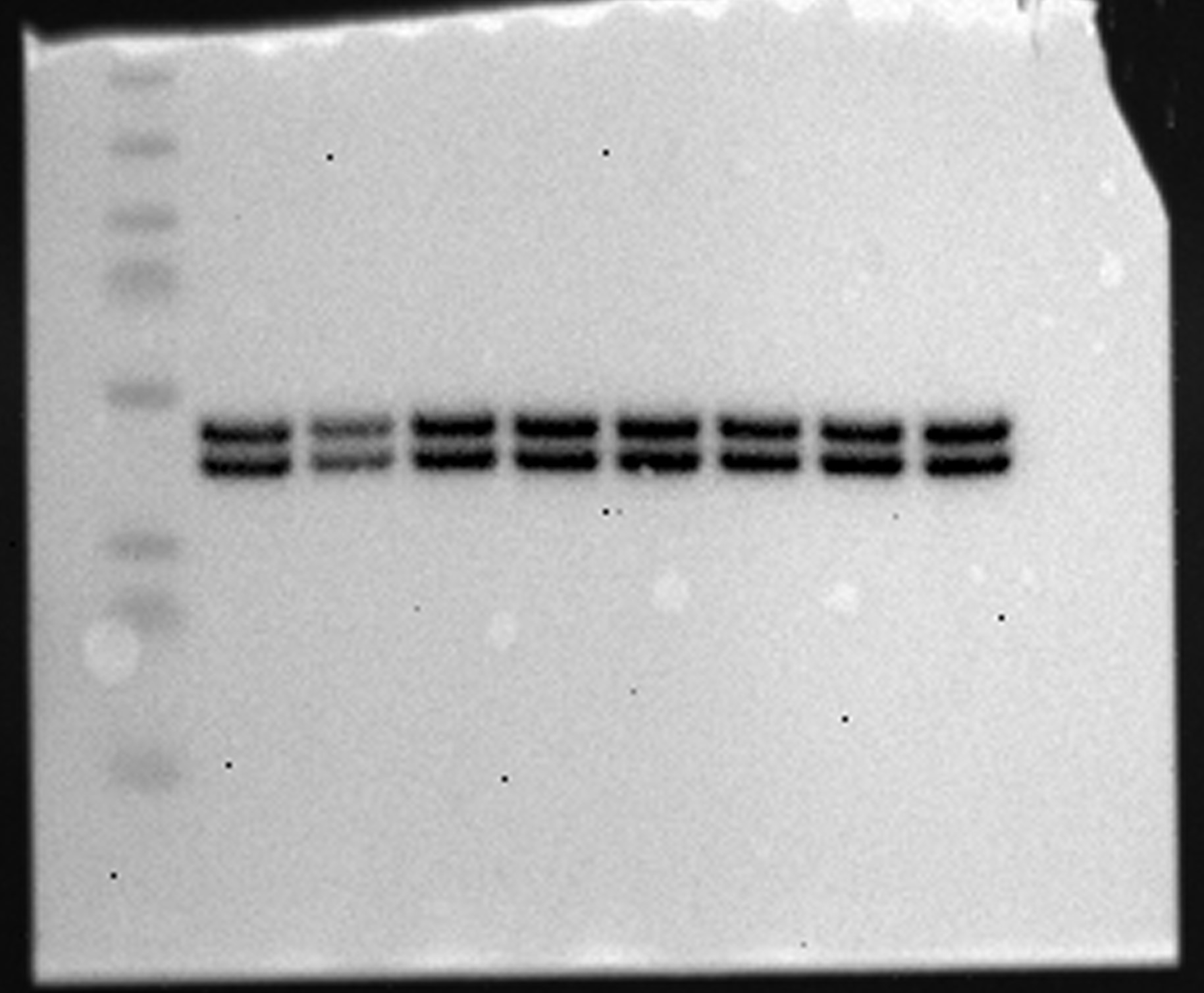

Supplement: Figure 1—source data 2. [file elife-94590-fig1-data2.zip › Figure 1A/erk total mb.jpg]

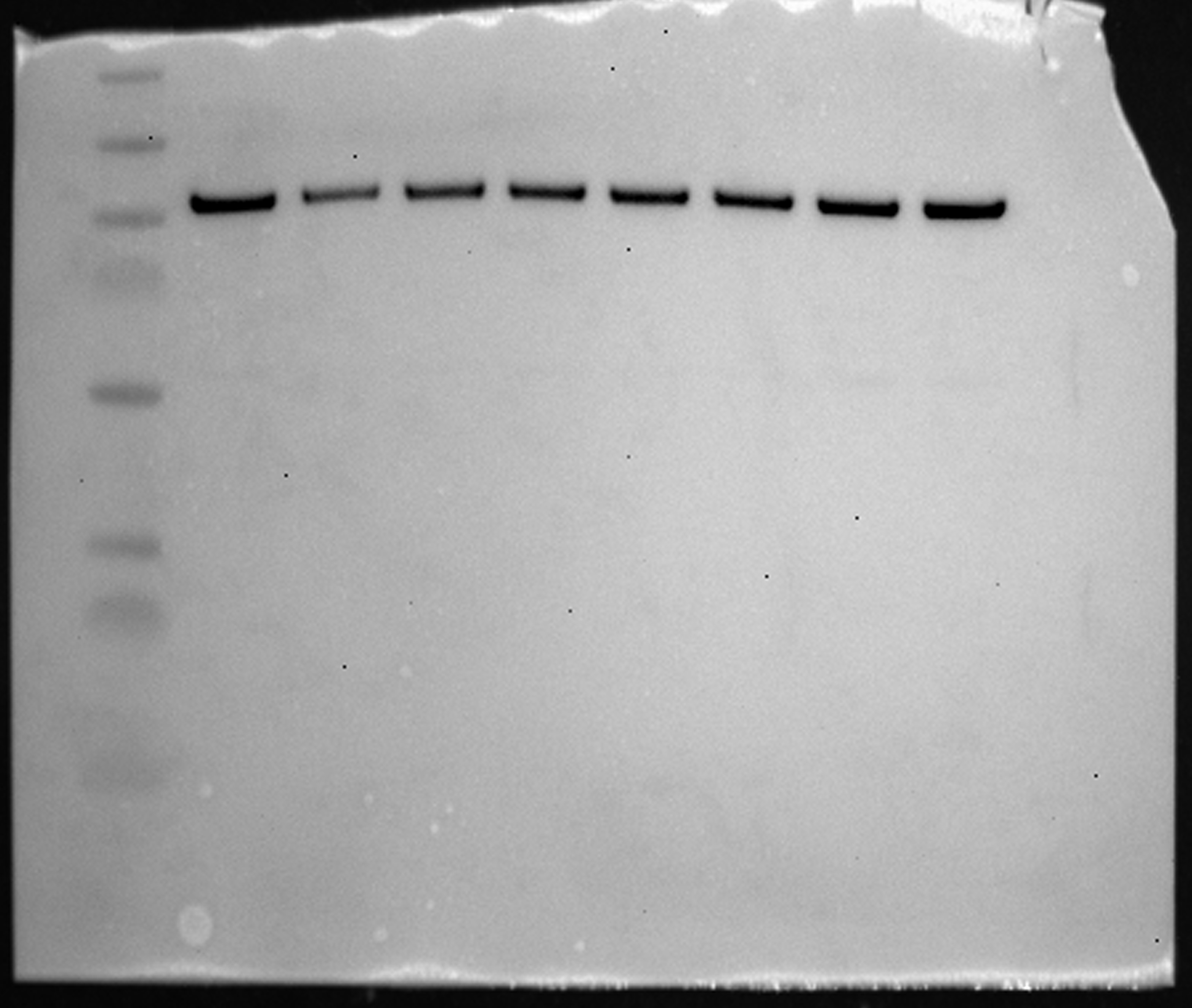

Supplement: Figure 1—source data 2. [file elife-94590-fig1-data2.zip › Figure 1A/HSP90 mb.jpg]

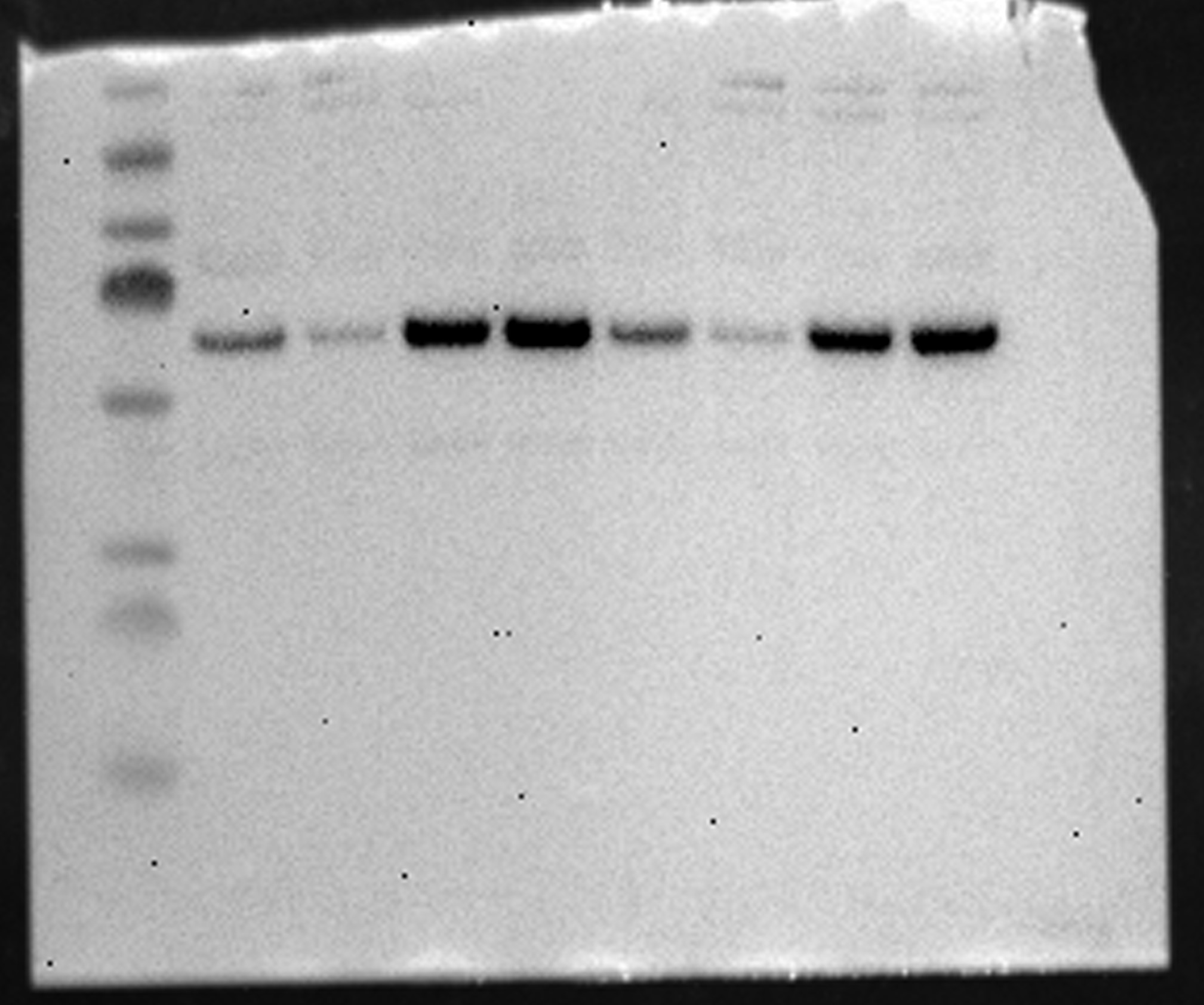

Supplement: Figure 1—source data 2. [file elife-94590-fig1-data2.zip › Figure 1A/pAKT mb.jpg]

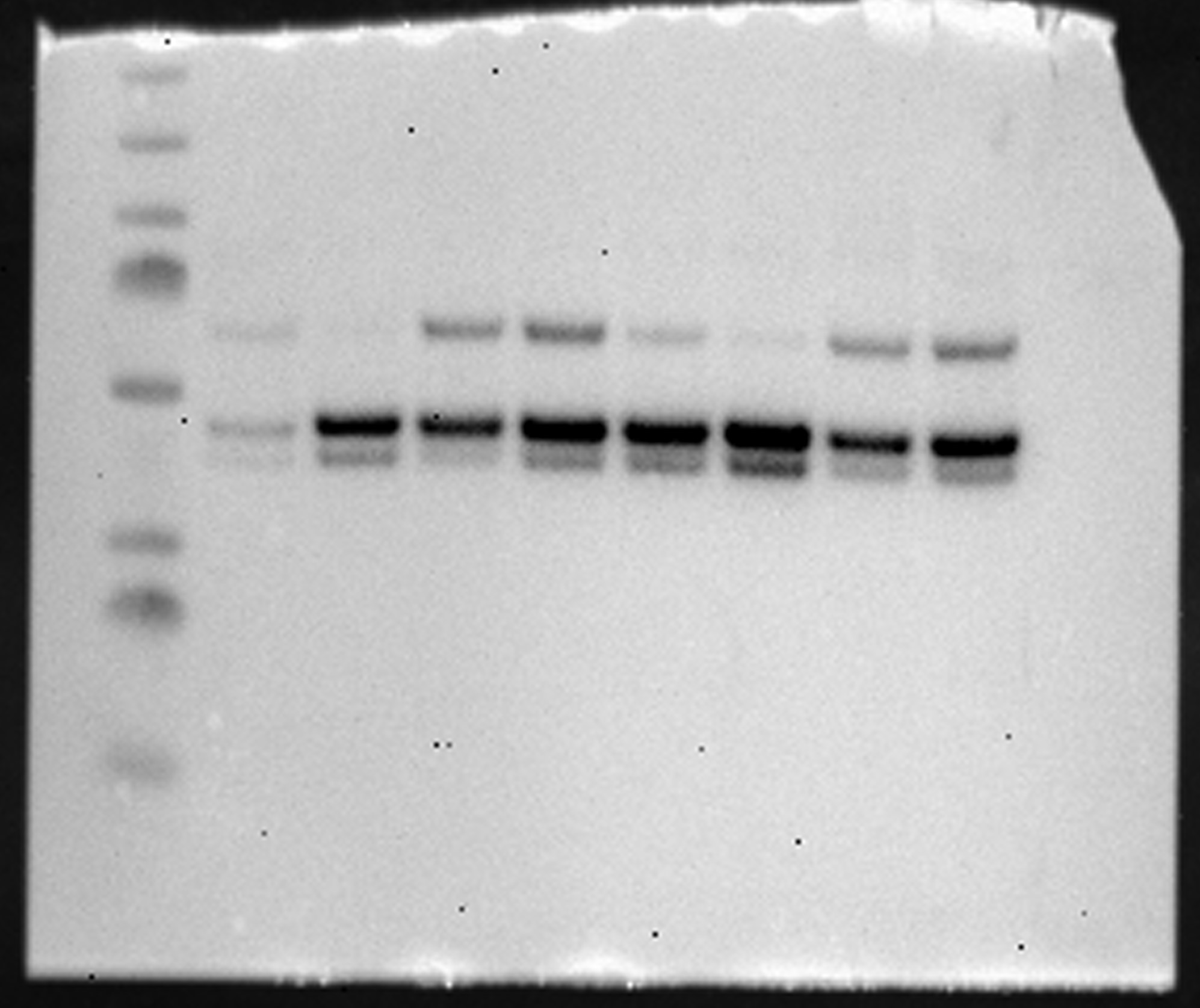

Supplement: Figure 1—source data 2. [file elife-94590-fig1-data2.zip › Figure 1A/pERK mb.jpg]

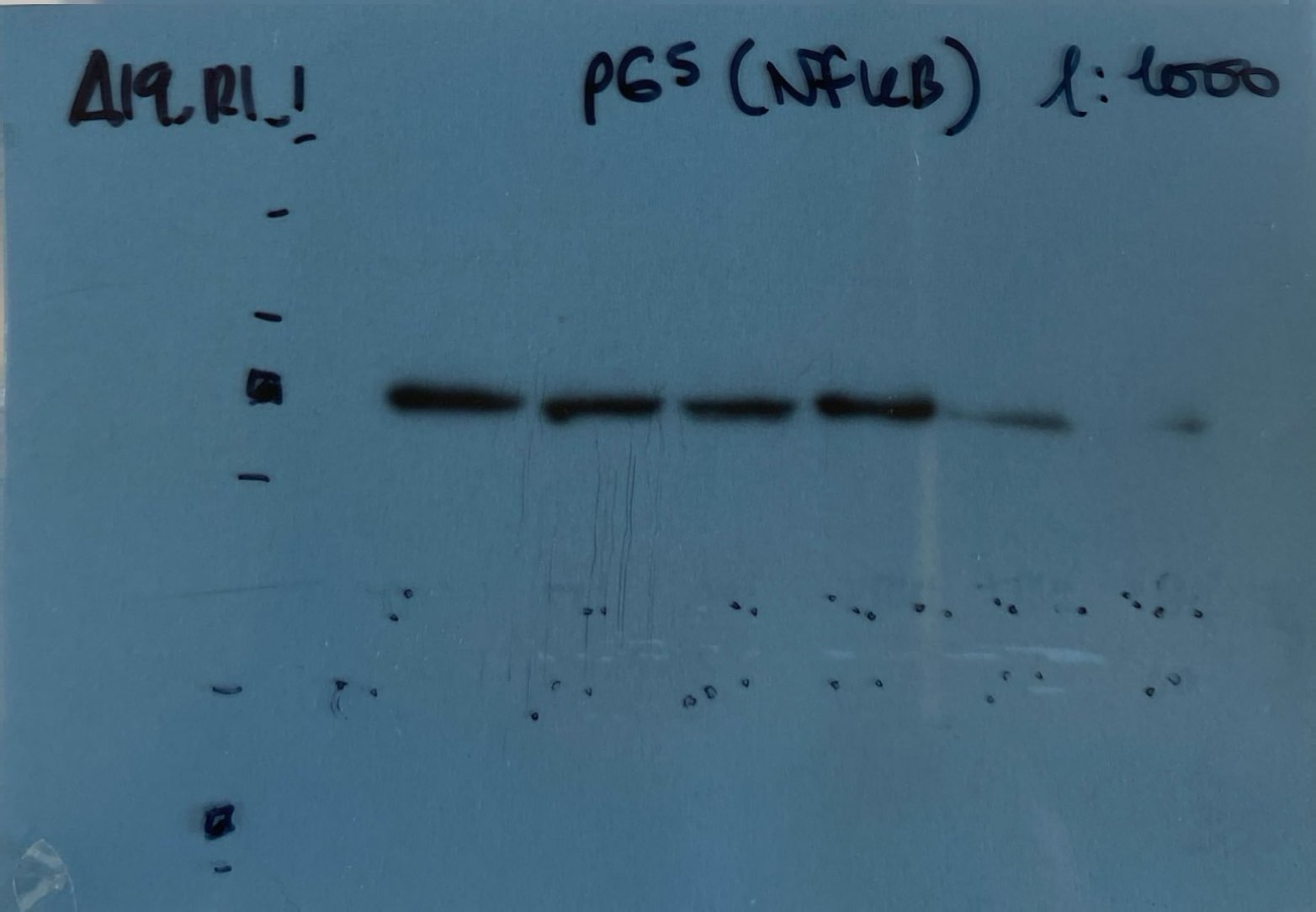

Supplement: Figure 1—source data 4. [file elife-94590-fig1-data4.zip › Figure 1B/p-p65 unlabelled.jpg]

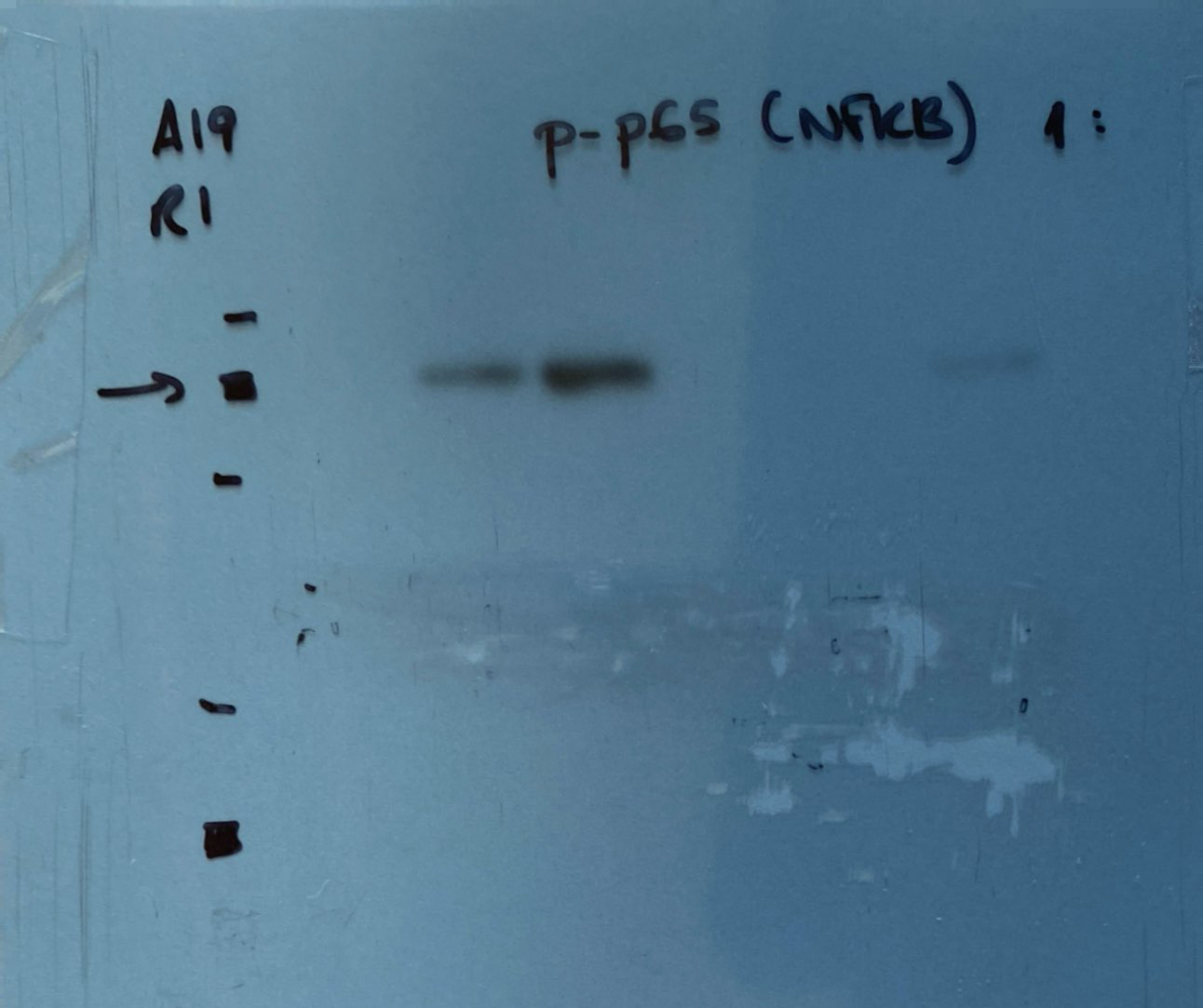

Supplement: Figure 1—source data 4. [file elife-94590-fig1-data4.zip › Figure 1B/p-p65_unlabelled.jpg]

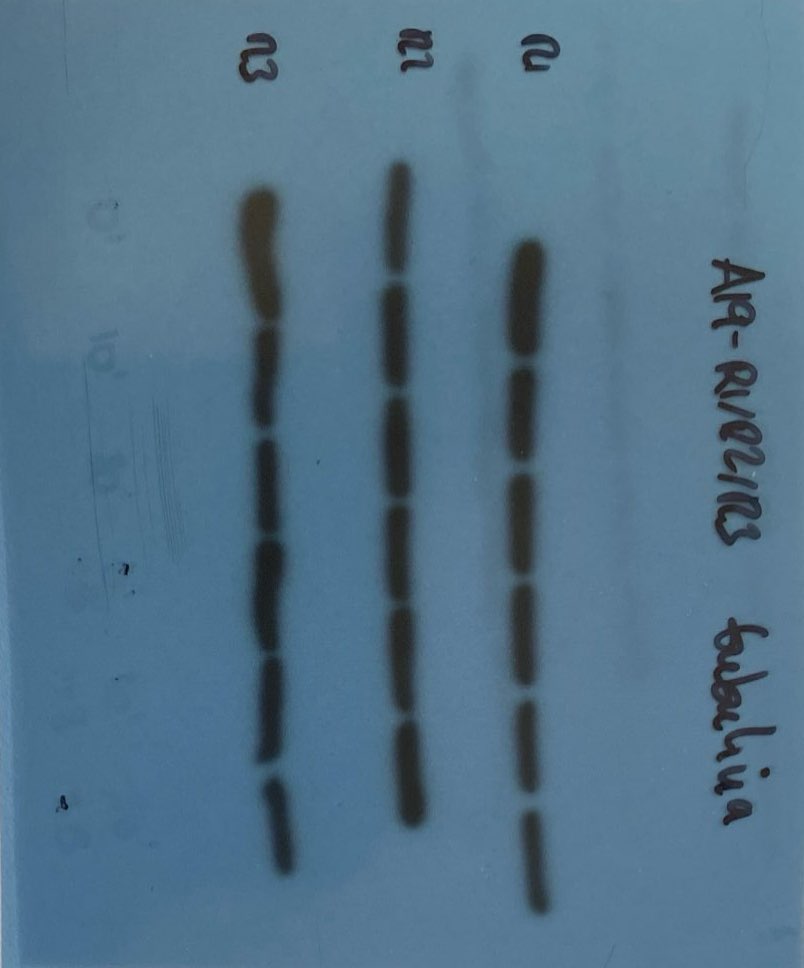

Supplement: Figure 1—source data 4. [file elife-94590-fig1-data4.zip › Figure 1B/Tubulin unlabelled.jpg]

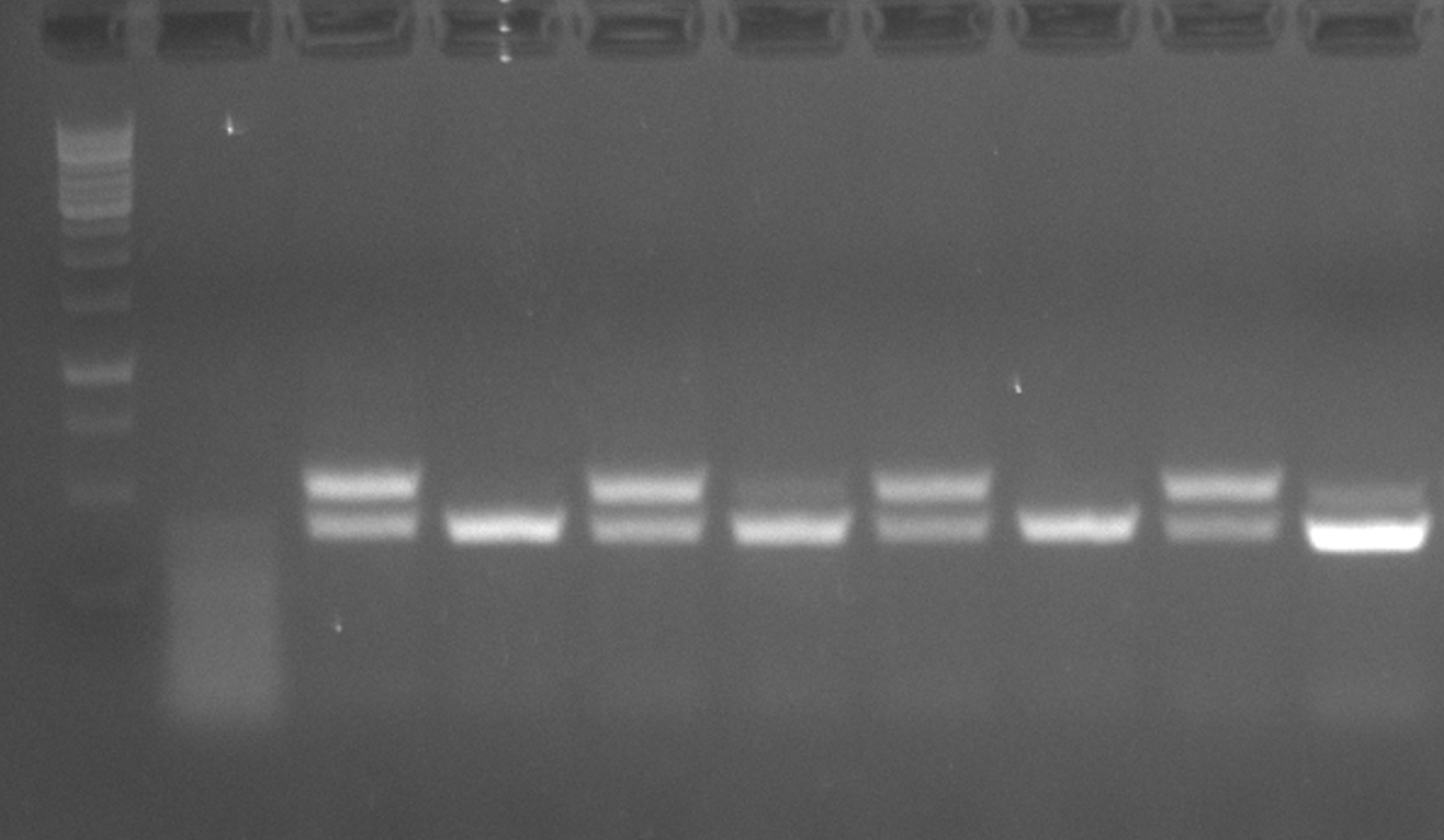

Supplement: Figure 1—figure supplement 1—source data 2. [file elife-94590-fig1-figsupp1-data2.zip › Figure 1-supplement 1B.jpg]

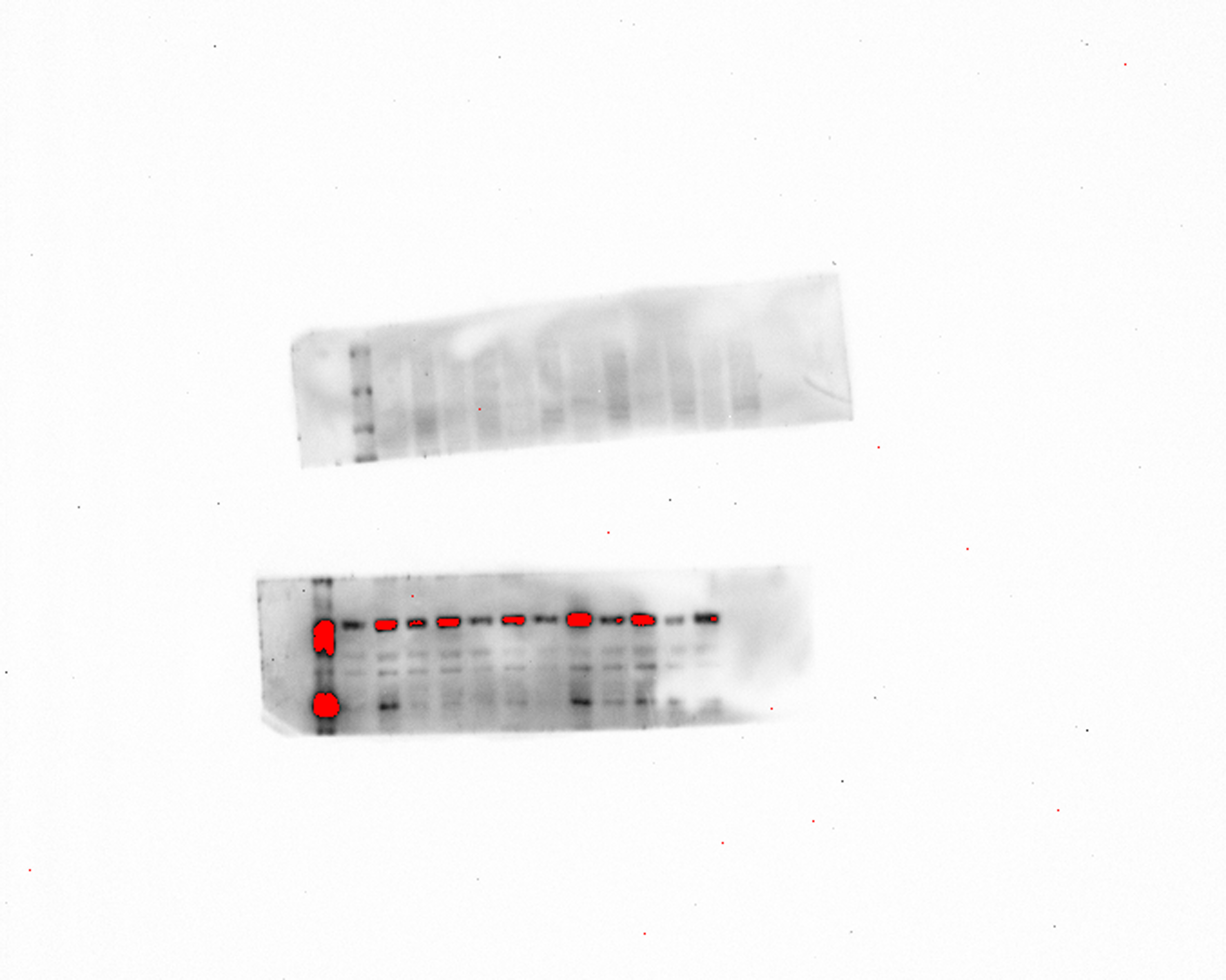

Supplement: Figure 6—source data 2. [file elife-94590-fig6-data2.zip › Figure 6-source data 2/Fig 6D Activated Cathepsin B.tif]

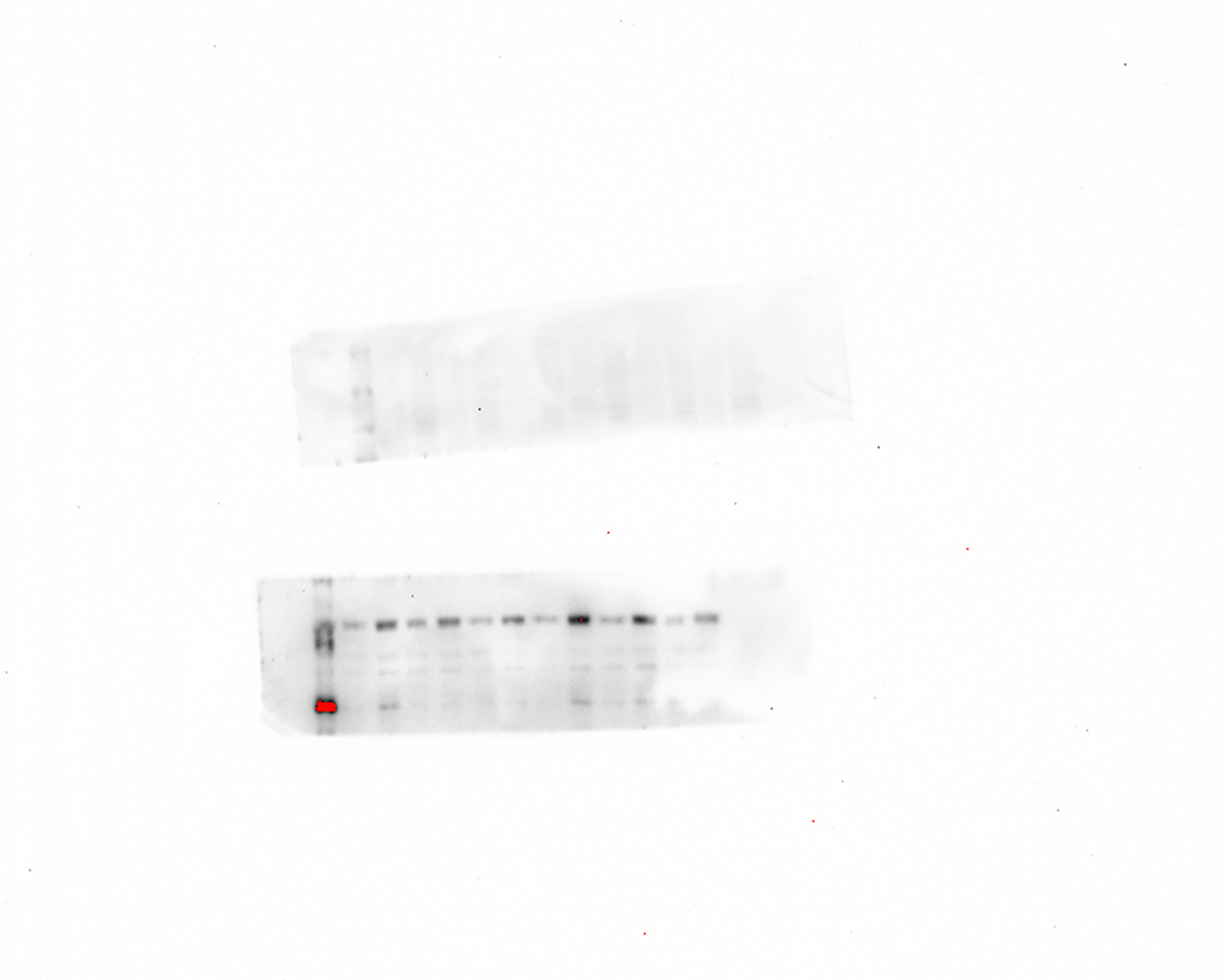

Supplement: Figure 6—source data 2. [file elife-94590-fig6-data2.zip › Figure 6-source data 2/Fig 6D Cathepsin B.tif]

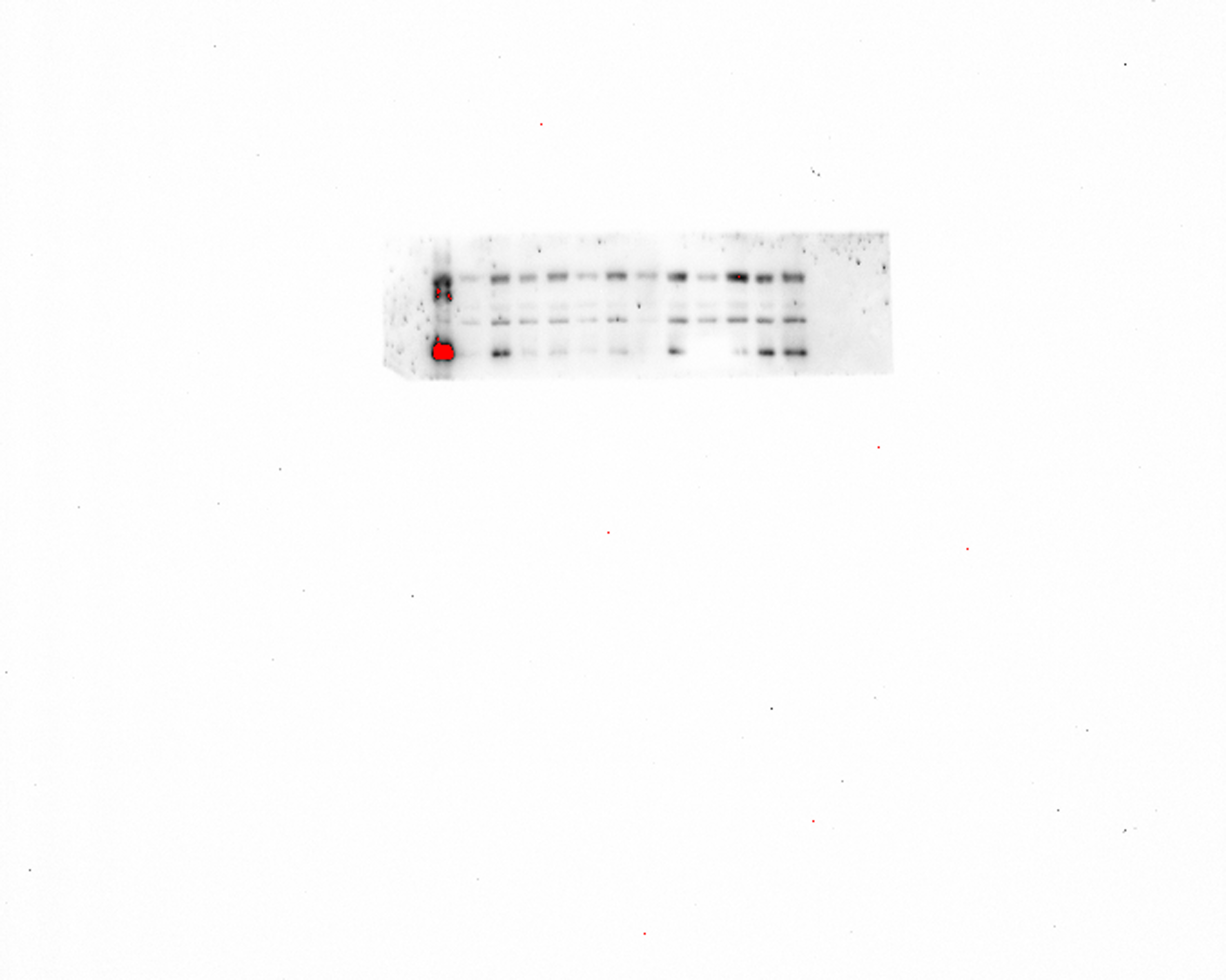

Supplement: Figure 6—source data 2. [file elife-94590-fig6-data2.zip › Figure 6-source data 2/Fig 6D Cathepsin D.tif]
