# Supplementary material for: RAS–p110α signalling in macrophages is required for effective inflammatory response and resolution of inflammation
Source: eLife. 2025 Apr 24;13:RP94590. doi: 10.7554/eLife.94590 (PMC12021417; doi:10.7554/eLife.94590)
Supplement: Figure 1—source data 3. [file elife-94590-fig1-data3.zip › Figure 1B-source data1.pdf]

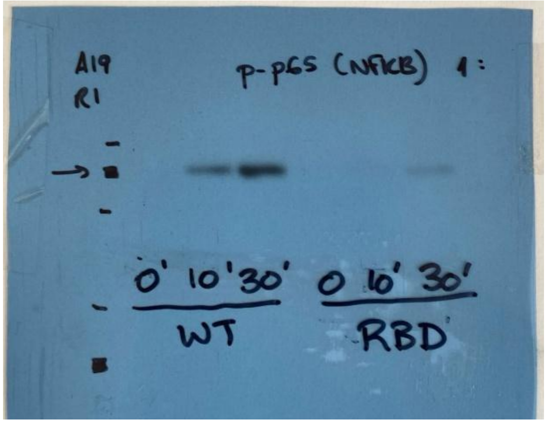

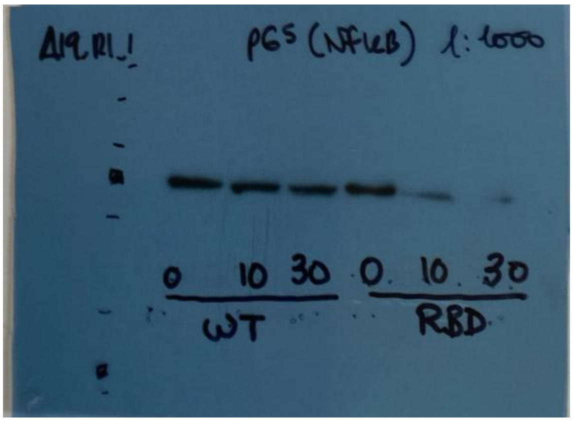

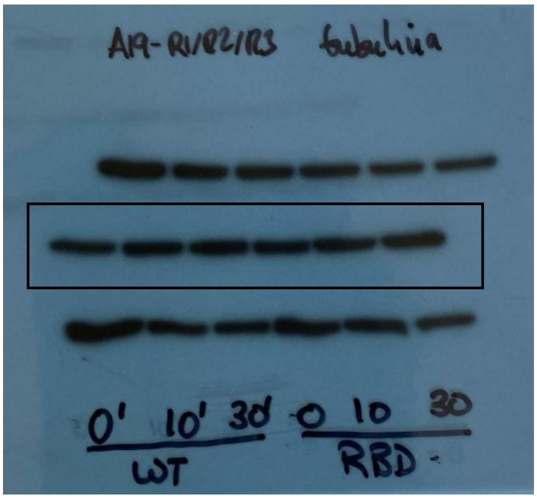

Figure 1B- Source data 1. Membranes corresponding to WB presented in Figure 1B. Original membrane was cut in two parts in order to incubate phosphor and total p65 in the upper band and tubulin in the lower part. Lower band was blotted with other samples.
