# Supplementary material for: RAS–p110α signalling in macrophages is required for effective inflammatory response and resolution of inflammation
Source: eLife. 2025 Apr 24;13:RP94590. doi: 10.7554/eLife.94590 (PMC12021417; doi:10.7554/eLife.94590)
Supplement: Figure 1—figure supplement 1—source data 1. [file elife-94590-fig1-figsupp1-data1.zip › Figure 1-supplement 1B.docx]

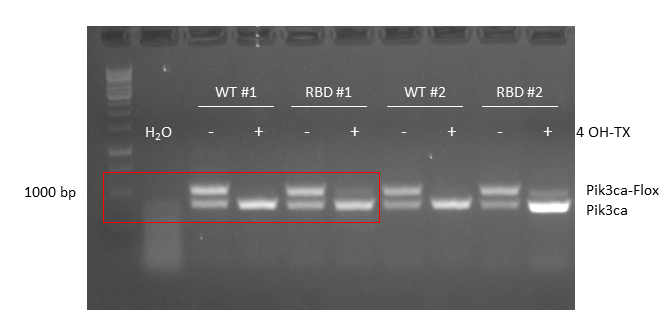


Figure 1-figure supplement 1B, source data 1: Original genotyping gel corresponding to Figure 1-figure supplement 1B. Red square indicates the samples that are shown in Figure 1-figure supplement 1B.
