# Supplementary material for: RAS–p110α signalling in macrophages is required for effective inflammatory response and resolution of inflammation
Source: eLife. 2025 Apr 24;13:RP94590. doi: 10.7554/eLife.94590 (PMC12021417; doi:10.7554/eLife.94590)
Supplement: Figure 6—source data 1. [file elife-94590-fig6-data1.zip › Figure 6D-source data 1.pdf]

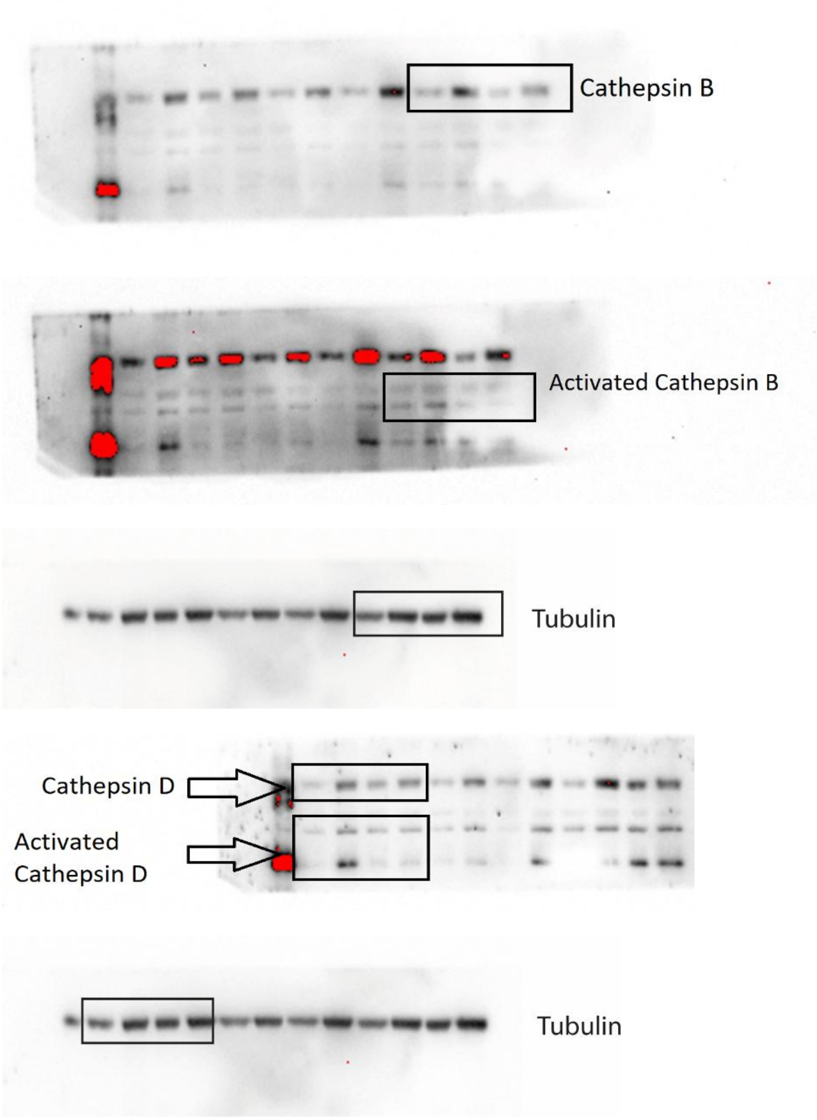

Figure 6D- Source data 1. Membranes corresponding to Cathepsin B, activated Cathepsin B, Cathepsin D, activated Cathepsin D and Tubulin (as loading control) Western Blots presented in Figure 6D. Samples used in this study are those marked in the rectangle. Sample order is (from left to right in the rectangle):

lane #1: WT BMDMs unstimulated

lane #2: WT BMDMs stimulated with LPS+IFN-γ

lane #3: RBD BMDMs unstimulated

lane #4: RBD BMDMs stimulated with LPS+IFN-γ
